# Exploring the Effects of a Brief Biofeedback Breathing Session Delivered Through the BioBase App in Facilitating Employee Stress Recovery: Randomized Experimental Study

**DOI:** 10.2196/19412

**Published:** 2020-10-15

**Authors:** Olga Chelidoni, David Plans, Sonia Ponzo, Davide Morelli, Mark Cropley

**Affiliations:** 1 Evolution, Behaviour and Environment School of Life Sciences University of Sussex Brighton United Kingdom; 2 Initiative in the Digital Economy Department of Science, Innovation, Technology, and Entrepreneurship University of Exeter Exeter United Kingdom; 3 Social Cognition Lab Department of Experimental Psychology University of Oxford Oxford United Kingdom; 4 BioBeats Group Ltd London United Kingdom; 5 Department of Engineering Science Institute of Biomedical Engineering University of Oxford Oxford United Kingdom; 6 Faculty of Health and Medical Sciences School of Psychology University of Surrey Guildford United Kingdom

**Keywords:** breathing, biofeedback, smartphone, heart rate variability, recovery, mindfulness, stress, mobile phone

## Abstract

**Background:**

Recovery from stress is a predictive factor for cardiovascular health, and heart rate variability (HRV) is suggested to be an index of how well people physiologically recover from stress. Biofeedback and mindfulness interventions that include guided breathing have been shown to be effective in increasing HRV and facilitating stress recovery.

**Objective:**

This study aims to assess the effectiveness of a brief app-based breathing intervention (BioBase) in enhancing physiological recovery among employees who were induced to cognitive and emotional stress.

**Methods:**

In total, we recruited 75 full-time employees. Interbeat (RR) intervals were recorded continuously for 5 min at baseline and during cognitive and emotional stress induction. The session ended with a 5-min recovery period during which participants were randomly allocated into 3 conditions: app-based breathing (BioBase), mindfulness body scan, or control. Subjective tension was assessed at the end of each period.

**Results:**

Subjective tension significantly increased following stress induction. HRV significantly decreased following the stress period. In the recovery phase, the root mean square of successive RR interval differences (*P*=.002), the percentage of successive RR intervals that differed by >50 ms (*P*=.008), and high frequency (*P*=.01) were significantly higher in the BioBase breathing condition than in the mindfulness body scan and the control groups.

**Conclusions:**

Biofeedback breathing interventions digitally delivered through a commercially available app can be effective in facilitating stress recovery among employees. These findings contribute to the mobile health literature on the beneficial effects of brief app-based breathing interventions on employees’ cardiovascular health.

## Introduction

Work environments have become more complex, and in the current scenario, there are incredible levels of job demands on employees. Increasing job demands are leading to unanimously negative trends toward occupational stress levels [1], which have been shown to result in damaging effects on employees’ health and well-being. High occupational stress levels are strongly associated with cardiovascular disease [2,3], which is one of the leading causes of morbidity and mortality [4].

Work stress may affect cardiovascular health through the imbalance between two major branches of the autonomic nervous system (ANS). The ANS consists of 2 antagonistic components: the sympathetic system, responsible for energy mobilization (fight or flight response), and the parasympathetic system, associated with restorative function (rest and digest status) [5]. Under optimal conditions, these systems operate in balance. However, under extended periods of work stress, the sympathetic system is overactivated, producing prolonged stress responses and eventually delaying stress recovery [6]. In the long term, if prolonged activation persists, it is thought to cause *wear and tear* on the physiological systems as higher levels of sympathetic activation lead to exhaustion [7]. What seems to be key for improving cardiovascular health is not the reactivity to stress but how quickly physiological recovery from stress occurs [8].

Heart rate variability (HRV) is a prominent biomarker of cardiovascular health that reflects the ability to maintain balance between sympathetic and parasympathetic activity in the presence of a stressor [9]. HRV refers to the time variation between interbeat (RR) intervals and HRV has been suggested to be an index of self-regulatory strength [10] and affective stability [11]. Higher HRV is a signal of good physiological adaptation, whereas lower HRV is an indicator of abnormal and insufficient adaptation of the ANS [12]. Individuals with naturally lower HRV have been shown to take longer to recover following exposure to experimentally induced stressors [13]. Low HRV has also been found to be a predictor of cardiac events [14], hypertension [15], and increased morbidity and mortality [16].

Given that HRV is a crucial index of somatic health and self-regulation, it is imperative to investigate possible ways to improve it, and growing evidence suggests that biofeedback is a simple and efficient way to increase HRV [17]. Biofeedback involves processes that enable individuals to learn how to change their physiological activity to improve health and performance [18]. One of these processes is to help individuals change their physiological responses by directly teaching them how to breath at a specific rate [19]. Respiratory sinus arrhythmia (RSA) is the mechanism through which breathing increases HRV. RSA occurs when heart rate fluctuations are synchronized with breathing; heart rate slightly increases during inhalation and slightly decreases during exhalation [20]. By teaching individuals breathing maneuvers, they learn to maximize and achieve RSA [19]. With breathing and heart rate at corresponding rates, the baroreflex, an ANS reflex responsible for controlling important bodily functions such as blood pressure (BP) [21], is strengthened. Thus, RSA not only increases HRV but also strengthens the baroreflex [22] and improves the control of physiological stress responses.

Previous research has shown that biofeedback interventions can be effective in increasing HRV. Hassett et al [23] showed that 10 biofeedback training sessions produced a significant increase in HRV among patients with fibromyalgia. Large and acute HRV increases were also observed after a 10-week biofeedback training among depressed adults [24]. Similar HRV increases were observed after 6 weeks of biofeedback training and maintained for 12 weeks after program completion in patients with coronary artery disease [25]. Empirical evidence suggests that biofeedback interventions can also result in significant improvements in self-reported stress and anxiety [26]. A study conducted among medical staff showed that a 28-day biofeedback training program led to significant decreases in self-reported work stress [27], and similar training has been successfully used as a stress reduction method for hospital nurses [28].

Furthermore, even brief biofeedback sessions can have positive effects in reducing stress reactivity in healthy populations. For example, it has been shown that participants who practiced 30 min of slow breathing demonstrated increased HRV during stress induction [29]. It has also been shown that a single session of biofeedback breathing can increase HRV [30], whereas a single 15-min breathing session can improve HRV in response to laboratory-induced stress and during recovery [31].

Beneficial effects on HRV can also be found following mindfulness-based interventions. Mindfulness is the practice of paying attention to the present moment in a nonjudgmental way by disregarding distracting thoughts and focusing attention on breathing [32]. Being trained to direct attention to a single focus such as breathing, while inhibiting any intrusive thoughts, can help individuals stay connected to their bodily senses [33]. A body scan is a common mindfulness practice that typically involves guided instructions where listeners attend to various parts of their body and their breathing, gently observing these areas and allowing other thoughts to recede [34]. Ditto et al [34] showed that participants practicing the body scan displayed significantly greater increases in HRV and larger BP decreases compared with those engaging in other relaxing activities. Shearer et al [35] also showed that students who underwent a one-day mindfulness training course that included breathing exercises demonstrated higher HRV in anticipation and during a cognitive challenge. More importantly, brief body scan sessions have also been shown to significantly reduce emotional exhaustion symptoms in health care workers [36].

At present, the mass popularity of smartphone devices opens possibilities for the use of mobile apps as a useful platform for delivering these self-help interventions. Their high accessibility and ease of use offer great potential for mobile health growth. Guided breathing can now be incorporated in mobile apps and delivered at a relatively low cost [37]. For example, previous research has shown that a biofeedback app-based game was more effective in decreasing arousal during stress when compared with conventional relaxation techniques [38], and an 8-week app-based mindfulness meditation program has also been shown to reduce stress in college students compared with controls [39].

Mobile apps have been used to modify stress responses, and recently there has been a surge in apps that aim to decrease workplace stress [40]. This is because many employees confront demanding tasks requiring high levels of cognitive effort on a daily basis. If tight deadlines and time pressure are added, then employees experience heightened levels of stress that elicit physiological arousal. It is also common for certain work situations involving high workload, heated interactions with supervisors, or discrepancies with colleagues to elicit negative emotions such as anger. These negative emotions, if not regulated efficiently, can trigger negative ruminative thoughts that, if persistent, can cause prolonged physiological activation [41], which in the long term can increase cardiovascular disease risk [16]. Therefore, it is imperative to find practical ways to help employees physiologically recover from the stress that work demands cause.

Most people who need such interventions are often those who are the most time deprived or busy. It is therefore important to develop parsimonious and easy-to-use interventions but also interventions that are evidence based. BioBase (BioBeats Ltd) is a commercially available mobile app used to modify breathing. Recently, breathing exercises delivered through BioBase were shown to produce significant HRV increases after stress exposure [42]. However, its effects on employee HRV have not been tested. This study therefore aims to examine the effects of a brief breathing session delivered through the BioBase app against a mindfulness body scan on HRV after stress exposure in a group of full-time employees. Participants were exposed to cognitive and emotional stress in the laboratory and then randomly allocated to 1 of 3 conditions: BioBase breathing app, mindfulness body scan, or a control condition.

It is intuitive that biofeedback and mindfulness share some degree of resemblance regarding the mechanisms they target; biofeedback improves breathing whereas mindfulness trains the individuals to focus their attention on bodily sensations such as breathing. Biofeedback is an active process that requires participants to achieve RSA by actively following specific audio and visual instructions and results in HRV changes very quickly [22]. However, a mindfulness body scan is a passive process involving audio instructions that require less active participant engagement, as its efficacy depends on participants’ attention levels and is generally considered to be effective only after regular practice [43]. Despite the abovementioned differences between biofeedback and mindfulness interventions, no study to date has investigated their effectiveness in comparison with one another and a control condition. Hence, the aim of this study was to assess the impact of a biofeedback intervention, namely guided breathing, in reducing stress, indexed by HRV recovery, in comparison with a mindfulness intervention and a control condition. As biofeedback requires participants to focus their attention on both audio and visual instructions, it was predicted that—following stress exposure—individuals in the BioBase breathing condition would demonstrate greater HRV recovery, relative to those in the mindfulness body scan condition.

## Methods

### Participants

In total, 107 individuals volunteered to participate. Of these 107 participants, 15 did not meet the eligibility criteria; 6 were receiving psychotherapeutic treatment, 3 had a heart condition, 1 took prescribed medication, and 5 were working less than 30 hours per week. Of the remaining 92, the first 75 individuals were tested, as this sample size has revealed moderate-to-strong effect sizes in the past [42]. The final sample consisted of 75 full-time working adults (48 females; age range 18-62 years; mean age 32.32, SD 10.46 years). Participants’ demographics such as work hours per week, level of education, years of work experience, and type of occupation are shown in Table 1. Single items were included asking participants if they were smokers, as frequent tobacco use has been associated with increased sympathetic activity resulting in higher heart rate (HR) [44]. Levels of fitness were also assessed as more fit individuals tended to have lower HR [45]. Smoking and fitness status for all participants is shown in Table 1. All participants were given a £10 (US $13) gift voucher for their participation. The University of Surrey Ethics Committee granted a favorable ethical opinion for this project (reference: UEC 2018 119 FHMS).

**Table 1:** Participant demographics (N=75).

Characteristics		Values, n (%)	
**Work hours per week**			
	31-40		65 (87)
	41-50		9 (12)
	>50		1 (1)
**Education**			
	No formal education		1 (1)
	Secondary education		13 (17)
	University education		61 (82)
**Work experience (years)**			
	Up to 1		33 (44)
	1-5		26 (35)
	>5		16 (21)
**Occupation**			
	Marketing or advertising		10 (13)
	Recruitment or Human Resources		9 (12)
	Teaching or education		9 (12)
	Accounting or banking or finance		7 (10)
	Business or consulting or management		6 (8)
	Engineering		5 (7)
	Health care		5 (7)
	Retail		5 (7)
	Research		4 (5)
	Other		15 (19)
**Smoking status**			
	Nonsmoker		52 (69)
	Past smoker		14 (19)
	Current smoker		9 (12)
**Fitness status**			
	Several times a week		34 (45)
	Once or twice a week		25 (33)
	Once or twice a month		8 (11)
	Less than once a month		5 (7)
	Never		3 (4)

### Design

This study was a randomized laboratory-based experiment that examined HRV changes following stress exposure. Initially, participants underwent a baseline period, followed by a stressor period, which included 2 cognitive tasks and 2 emotion-eliciting film clips. They were then randomly assigned to 1 of the 3 conditions: BioBase app, mindfulness body scan, or control. HRV was assessed throughout the experiment.

### Cognitive Stress

Cognitive performance-based tasks require high cognitive effort, and they increase physiological activation (HR) [46]. The use of nonverbal tasks also avoids the effect of cardiovascular confounds such as speaking, and its effects on respiration during a verbal stress task can mask the acute effect of the physiological stress response on HRV [47]. Therefore, in this study, we administered 2 tasks to induce cognitive stress. The first was the Continuous Performance Task, a neuropsychological performance-based task that assesses sustained attention. In this task, participants had to attend a series of letters presented on the screen and respond by pressing the space bar whenever an “X” was presented. Letters were presented in 9 blocks of 20 letters, 5 of which were “X” in each block. All letters were displayed for 250 ms each. The interstimulus intervals were 1 second, and participants were given a *practice round* before commencing to make sure they understood when they needed to respond. The task lasted approximately 8 min and was adapted from a previous study [48]. The second task we used was the stop signal paradigm of Go/No-Go, which assesses inhibition. Participants were presented with a series of images. About 75% of these images were acting as Go *cues*, and participants needed to respond (press the space bar) as fast as possible. Occasionally the series of Go *cues* got interrupted by images that were acting as No-Go *cues*, and participants were instructed not to respond. Owing to the frequency of the Go *cues* (≥75%) being greater than the No-Go *cues*, a tendency to respond is created while participants should effortfully inhibit their responses to the No-Go *cues*. Participants responded to 2 blocks of 80 trials each, and this task was adapted from a previous study [49]. Participants were told to answer as quickly as possible in both tasks, which were programmed using E-PRIME 2.0(Psychology Software Tools).

### Emotional Stress

Emotional stress was induced by asking participants to watch 2 emotion-eliciting film clips, a common technique to induce emotions in the laboratory while maintaining ecological validity [50]. The film clips were derived from the FilmStim database [51], which includes videos for emotion elicitation purposes. Participants watched 2 anger-eliciting film clips because anger has been found to induce the greatest physiological activation in HR among negative emotions [52]. The first anger-eliciting film clip was a 93-second excerpt from the film “American History X” which was found to produce a high arousal score (5.84/7) and the maximum negative affect score among databases (2.73/5). The second was a 43-second excerpt from the film “The Piano,” producing a high arousal score (5.67/7) and very high negative affect score (2.49/5) [51]. Sound from both clips was removed to ensure that participants’ attention was focused on the images [53].

### BioBase App

The BioBase app is a commercially available app that provides guided breathing that can be modified according to participants’ HR. In this study, participants in the BioBase breathing condition performed a 5-min guided breathing exercise based on the Papworth-Benson breathing method, which focuses on diaphragmatic breathing to use the full lung capacity and aims to decrease breathing rate, which will then trigger vagus nerve stimulation, leading to a desired relaxation response [42]. The relaxation phase occurs when HR and breathing synchronize or become resonant. Participants in this condition followed visual and audio instructions on a smartphone without being given feedback on their breathing. Instructions guided participants to breathe at 6 breaths per minute for 5 min. This is a common breathing rate that results in great HRV increases, which can occur within the first few minutes of practice [54].

### Mindfulness Body Scan

Participants in the mindfulness condition were asked to perform a body scan for 5 min by following audio instructions [55]. This required participants to focus on the audio guiding their attention on their bodily and breathing sensations and encouraging nonjudgmental acceptance of thoughts and feelings experienced at that moment.

### Self-Report Measures

#### Mindfulness

Participants completed the Five Factor Mindfulness Questionnaire (FFMQ) [56] before the experiment. The FFMQ is a 24-item self-report measure that assesses 5 distinct but related facets of mindfulness and consists of 5 different subscales: nonreactivity to inner experience, observing or noticing, acting with awareness, describing, and nonjudging of experience. Participants responded on a 5-point Likert scale ranging from 1 (never or very rarely true) to 5 (very often or always true).

#### Fatigue

Fatigue makes it difficult to maintain attention and has been found to impair performance in attention tasks [57]. To ensure that participants would be able to focus on the cognitive stressor task, we further controlled for fatigue by using the Samn-Perelli Fatigue Checklist [58] before testing started. This single item assesses state fatigue by asking participants to rate their fatigue levels on a 7-point Likert scale (1=fully alert to 7=completely exhausted, unable to function effectively, ready to drop).

#### Sleepiness

We also controlled for sleepiness levels as sleep deprivation is known to degrade inhibitory control [59]. The Stanford Sleepiness Scale [60] was used to assess subjective perceptions of daytime sleepiness/alertness by asking participants how sleepy/alert they felt before the experiment on a 7-point Guttman scale item (1=feeling active and vital, alert, wide awake to 7=lost struggle to remain awake).

#### Mood

The Visual Analog Scale (VAS) for Moods [61] was used as a brief measure to repeatedly assess participants’ mood changes after the completion of each period. Participants were asked to indicate how tense they felt after the baseline period, the cognitive stressor, the emotional stressor, and the recovery period. Participants were asked, “At this particular moment, how tense do you feel?” and they answered by moving a marker up and down a 100-mm line representing a continuum from 0 (extremely tense) to 100 (not tense at all). Higher scores indicate higher levels of tension.

### Heart Rate Variability

A Polar V800 HR monitor with a Polar H7 HR Sensor (Polar Electro OY) was used to record RR intervals at a sampling frequency of 1000 Hz [62]. The HR sensor was attached to a chest band, which sends a signal via Bluetooth to a smartwatch. RR intervals were continuously recorded throughout the experiment.

### Procedure

**Figure 1:**
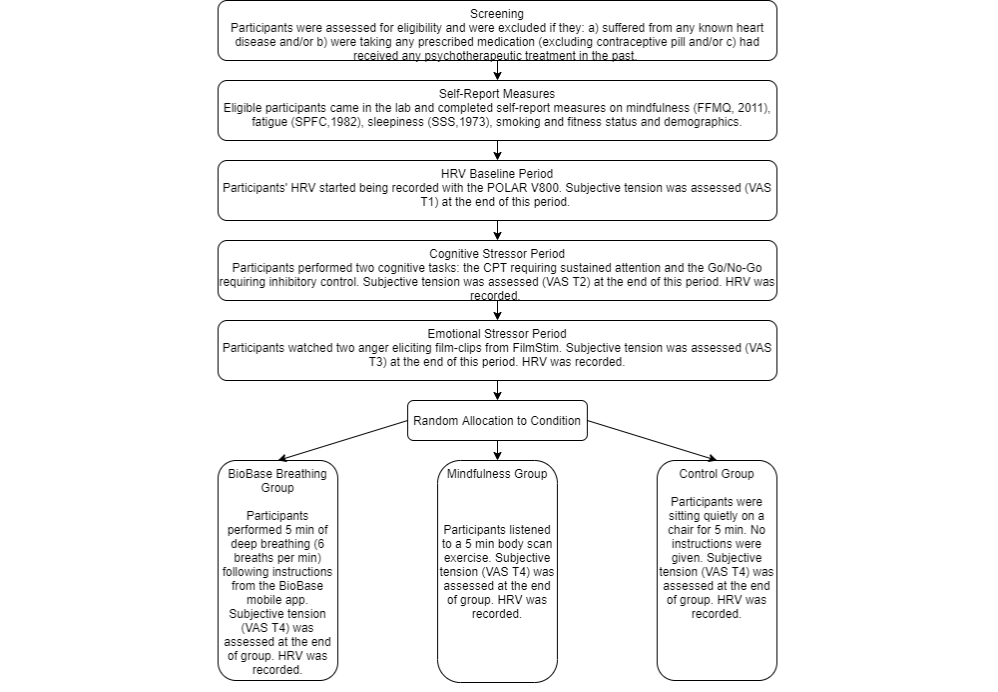
Procedure flow chart. CPT: Continuous Performance Task; FFMQ: Five Factor Mindfulness Questionnaire; HRV: Heart Rate Variability; SPFC: Samn-Perelli Fatigue Checklist; SSS: Stanford Sleepiness Scale; VAS: Visual Analogue Scale.

The study was conducted in a laboratory at the University of Surrey. Before attending the laboratory session, participants gave their informed consent and completed a short health screening survey. Participants who reported having any heart condition or taking any prescribed medication (not including contraceptive pill) were excluded from participation as some pathological heart-related conditions such as arrhythmia and various types of medication can confound the interpretation of HRV values [63]. Those who reported having received psychotherapeutic treatment in the past were also excluded as we wanted to ensure that the conditions were novel to participants. In the laboratory, participants completed self-report measures on their demographics and control variables reported in the *Self-Report Measures* section. They were then given instructions on how to place the HR chest band. The experimenter left the room to allow privacy for participants to place the chest band. Once the chest band was safely attached, and HR readings were successfully synchronized with the smartwatch, participants were asked to quietly remain seated on a chair for 5 min; this was the baseline period. Once the 5 min had passed, they were asked to indicate how tense they felt by completing the VAS. Following this, they were asked to complete 2 cognitive tasks on the computer. Participants were given clear instructions on how to complete the tasks and were told that they measured attention speed and they had to respond as quickly as possible. To ensure that they understood the instructions, they completed a short practice block for which they were given feedback on their performance before each task. Once participants completed the tasks, they were asked to indicate how tense they felt by completing the VAS. They were then told that they had to watch 2 short film clips on the computer. They were not aware of the film clips’ emotional valence. Once the film clips were viewed, participants were again asked to indicate how tense they felt on the VAS. Participants were then randomly allocated to 3 different recovery conditions: BioBase breathing, mindfulness body scan, and control. Block randomization was used to allocate participants into 3 groups, and web-based software was used to generate the group allocation. Participants in the control condition were asked to remain seated silently on the chair. They were not given any specific instructions regarding their breathing rates. At the end of the 5-min recovery phase, participants across all 3 conditions were asked to indicate how tense they felt on the VAS. Finally, they were told that the experiment had ended and the HR recording was turned off. Participants were asked to remove the chest band. The experimenter left the room and returned once participants were ready, thanked them for their participation, and gave them the debrief statement. Figure 1 shows the flowchart of the procedure.

### Data Analysis

**Figure 2:**
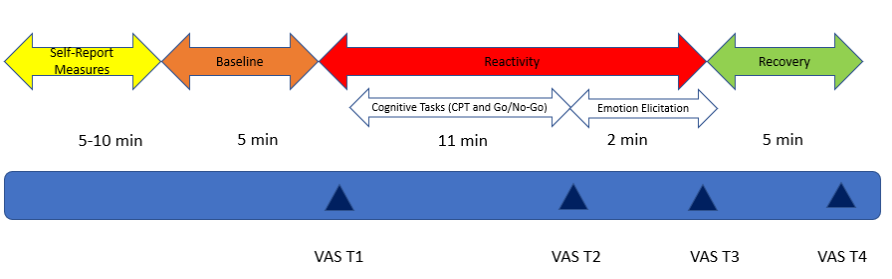
Experiment timeline. CPT: Continuous Performance Task; VAS: Visual Analogue Scale.

The HRV analysis was conducted using ARTiiFACT software [64]. General guidelines [65] considered 5-min excerpts as an accepted short-term recording for group comparison and a minimum of 250 seconds is suggested for organizational research [66]. For this study, RR excerpts were grouped into 4 epochs (baseline, cognitive stressors, emotional stressors, and recovery). The baseline period was analyzed in 5-min segments; the cognitive stressor, in 11 min; the emotional stressor, in 2 min segments; and the recovery period, in 5-min segments across all participants. For the data analysis, values from the cognitive and emotional stressor periods were averaged and therefore analyzed as one total stressor period. Following previous research [42,67], we also accounted for individual differences in stress responsivity that could potentially influence the findings by creating a stress reactivity change score (derived from calculating the HRV difference between the total stressor period and the baseline period). To reduce HRV artifacts (owing to movement, sneezing, or loss of signal), recordings were taken while participants were sitting with knees at a 90^o^ angle, both feet flat on the floor without speaking or making any movements [68]. Artifacts were identified using the Berntson detection algorithm. Flagged RR intervals were visually inspected, and once confirmed as artifacts, they were corrected by applying cubic and/or linear spline interpolation, where necessary [69]. For this study, we used 3 HRV parameters. The first parameter was the root mean square of successive RR interval differences (RMSSD), which is a time-domain measure considered to reflect vagally mediated HRV. The second parameter was percentage of successive RR intervals that differ by more than 50 ms (pNN50), which reflects the percentage of successive RR intervals that differ >50 ms. The third parameter was the high frequency (HF), a frequency-domain parameter that represents breathing-related changes in HR, reflects parasympathetic contribution to HRV, and is regarded as an RSA measure [70]. Before analyses, the data were screened for outliers and normality. All HRV variables were positively skewed; therefore, data were log-transformed (lg10) because this transformation type can correct for positive skewness and kurtosis, unequal variances, and lack of linearity [71]. On the basis of their z-scores, 3 outliers were excluded as they were above 3 SDs. Statistical analyses were performed using SPSS 25. Figure 2 shows the experiment timeline.

## Results

### Participant Characteristics

One-way analysis of variances (ANOVAs) were conducted to detect any between-group differences (BioBase, mindfulness, and control) regarding baseline HRV. There were no significant differences between groups regarding baseline HRV as reflected on root mean square of successive RR interval differences (RMSSD; *F*_2,72_=0.42; *P*=.65; *η^2^*=0.01), pNN50 (*F*_2,69_=1.42; *P*=.24; *η^2^*=0.04)*, and HF* (*F*_2,72_=2.12; *P*=.33; *η^2^*=0.05). We also tested for between-group differences in demographic variables such as age, gender, work hours, and external factors such as smoking, fitness status, and trait mindfulness to determine whether they had a systematic effect on HRV. Participant characteristics are presented in Multimedia Appendix 1. One-way ANOVAs on levels of fatigue (*F*_2,72_=1.40; *P*=.25; *η^2^*=0.03) and sleepiness/alertness (*F*_2,72_=1.27; *P*=.28; *η^2^*=0.03) revealed no significant difference between participants.

A paired sample *t* test was conducted to demonstrate that the experimental manipulation was successful (cognitive tasks and anger-eliciting film clips). Subjective tension after the cognitive task and the emotional task were averaged and then compared with subjective tension at baseline. RMSSD values after the cognitive task and emotional task were also averaged. We did this to capture HRV during the total reactivity period (Figure 2). Results showed that subjective levels of tension significantly increased after the cognitive and emotional stressors (mean 4.21, SD 2.18) compared with baseline (mean 1.28, SD 1.66), t_74_= −10.84, *P*<.001, *d*=0.94. Similarly, RMSSD during the stressor period was significantly lower (mean 1.60, SD 0.23) than the baseline RMSSD (mean 1.68, SD 0.27), t_74_=3.39, *P*=.001, *d*=0.48, indicating that the experimental manipulation was successful in physiologically arousing participants.

### Poststress Physiological Recovery

To examine the effects of the intervention on cardiac recovery in the poststress period, a between-subjects analysis of covariance for the group (BioBase, mindfulness body scan, and control) was conducted using the HRV at recovery as the outcome and the reactivity change score as a covariate (to statistically remove its influence on recovery). Age was also added as a covariate as responsiveness of autonomic activity reduces with older age [72]. There was a significant group effect for RMSSD, pNN50, and HF with a medium-sized effect, regardless of participants’ individual differences in stress response. In all cases, HRV was higher in the BioBase group than in the mindfulness body scan and the control group. The results are shown in Table 2.

**Table 2:** Group means, standard deviations, between-group F ratio and effect sizes in the BioBase breathing, mindfulness body scan, and control group during poststress recovery.

Parameters	Groups			Values		
	BioBase breathing, mean (SE)	Mindfulness body scan, mean (SE)	Control, mean (SE)	*F* test (*df*)	*P* value	ηρ^2^
RMSSD^a,b^	1.82 (0.05)	1.68 (0.05)	1.55 (0.05)	6.801 (2,72)	.002	0.16
pNN50^a,c^	1.37 (0.07)	1.29 (0.07)	1.03 (0.08)	5.261 (2,69)	.008	0.13
HF^a,d^	2.97 (0.10)	2.77 (0.11)	2.50 (0.11)	4.695 (2,72)	.01	0.12

^a^Log transformed.

^b^RMSSD: root mean square of successive interbeat interval differences.

^c^pNN50: percentage of successive interbeat intervals that differ by more than 50 ms.

^d^HF: high frequency.

## Discussion

### Principal Findings

It is common for employees to encounter stressors at work that can cause physiological activation and, if prolonged, can in the longer term have deleterious effects on cardiovascular health [41]. The aim of this study was to compare the effects of a 5-min guided breathing exercise delivered through the BioBase app with a mindfulness body scan exercise and a control group in facilitating employees’ physiological recovery following induced stress exposure.

Our principal finding was that HRV was higher at recovery for participants who performed BioBase breathing after stress induction. Results showed that there was a significant difference between groups, with those performing biofeedback breathing on the BioBase app demonstrating higher HRV compared with mindfulness and the control group during the recovery period. This is of great significance because HRV is an important cardiovascular health index, and higher HRV has been associated with numerous positive health outcomes [73].

### Comparison With Previous Work

Recent studies have also investigated the effectiveness of app-based biofeedback breathing interventions. For example, Plans et al [42] assessed the effects of 6-min guided breathing delivered through the BioBase app compared with 6 min of rumination and controlled conditions after stress exposure induced by completing a speech task. They found that individuals who performed guided breathing on the BioBase app had significantly higher HRV than those who ruminated and the control group poststress. Our study findings are in line with their findings, but more importantly, this study was designed to overcome some of their limitations. For example, their study did not account for confounding variables such as fitness and smoking status. Furthermore, they compared the effects of breathing with rumination, which mostly pertains to repetitive thinking of negative nature, whereas we compared breathing with mindfulness, an active intervention historically associated with positive health outcomes. In addition, the breathing rate used in this study was based on past literature suggesting 6 breaths per minute as the average rate to achieve RSA [54]. Our findings also support recent evidence showing that a brief 5-min app-based biofeedback training session was effective in enhancing physiological recovery after laboratory-induced stress, as assessed by levels of salivary alpha-amylase, another commonly used stress biomarker [74].

Previous studies have compared the effects of biofeedback breathing and mindfulness with other self-help methods. For example, a study compared the effects of a 5-week daily biofeedback breathing intervention with mindfulness and physical activity training [75] and reported no overall difference in their effectiveness. Biofeedback training was also found to be equally effective with mindfulness and physical exercise training in reducing worry symptoms and improving attention in young adults [76]. These results are in contrast with our findings, which revealed that biofeedback breathing resulted in greater increases in HRV compared with mindfulness. However, it should be noted that these previous studies [75,76] are limited by the fact that the interventions consisted of numerous sessions, and outcome measures were of a self-report nature while our study looked at physiological changes over a single session. Moreover, these previous studies examined the intervention effects on stress responses, whereas this study examined the effect of the intervention on recovery from physiological stress. Further research is needed to shed light on the differential effects that brief interventions and long-term interventions can have on physiological and self-report outcome measures.

### Limitations

Despite our study building upon and extending findings from the previous studies, it comes with its own limitations that warrant discussion. First, HRV during recovery was recorded for only 5 min. Even though this is classified as the minimum accepted short-term recording [65], it could be of great interest to explore how long after the recovery period did the effects last. Moreover, we induced anger among participants, which is a commonly experienced emotion in the workplace that can pose detrimental effects not only for the employee experiencing it but also on the organizational level [77]. Therefore, our results add value to the notion that BioBase was successful in helping employees physiologically recover from a negative emotion. However, it would be worth exploring the effectiveness of the BioBase breathing app in helping employees overcome negative emotions other than anger. Another limitation is that we did not establish the resonant frequency for each individual but we used the commonly accepted average of 6 breaths per minute. Lehrer et al [54] suggested that practicing breathing at the unique resonant frequency for each individual is a requirement to produce maximal HRV increases. Our study included a single brief breathing session, so establishing each participant’s resonant frequency would be time consuming. Greater effect sizes might have emerged if we applied the unique resonance frequency breathing rate for each participant.

Regardless of these limitations, the results derived from this study provide a stimulus for the delivery of future stress recovery interventions. It would be of interest to test the effects of repeated biofeedback breathing exercises on employees’ health. For example, future studies could investigate whether the daily practice of short biofeedback sessions could potentially result in HRV increases not only during practice but also in trait physiological changes in HRV. It would also be of interest to test whether biofeedback breathing could increase HRV among employees who ruminate about work during their leisure time [78].

### Conclusions

To our knowledge, this study is the first randomized study to investigate the effect of biofeedback and mindfulness on physiological recovery from momentary stress in employees. This study demonstrated that a short period of focused biofeedback breathing can aid physiological recovery in employees following a laboratory-induced stressor. Using a smartphone app to aid physiological relaxation may also help individuals to recover from stressful situations in real life, both inside and outside of work. By taking into consideration the rapid advances in mobile technology, this study showcases the great utility of short smartphone-delivered relaxation methods. With their great practicality and high accessibility, mobile interventions can be a potential solution to the ever-increasing demand for ease of use and parsimonious stress-management interventions.

## Appendix

Multimedia Appendix 1 Participants’ characteristics per group.

## Abbreviations

ANOVA: analysis of variance
ANS: autonomic nervous system
BP: blood pressure
FFMQ: Five Factor Mindfulness Questionnaire
HF: high frequency
HR: heart rate
HRV: heart rate variability
pNN50: percentage of successive RR intervals that differ by more than 50 ms
RMSSD: root mean square of successive RR interval differences
RR: interbeat
RSA: respiratory sinus arrhythmia
VAS: Visual Analog Scale
