# Supplementary material for: Exploring the Effects of a Brief Biofeedback Breathing Session Delivered Through the BioBase App in Facilitating Employee Stress Recovery: Randomized Experimental Study
Source: JMIR Mhealth Uhealth. 2020 Oct 15;8(10):e19412. doi: 10.2196/19412 (PMC7596654; doi:10.2196/19412)
Supplement: Multimedia Appendix 1 [file mhealth_v8i10e19412_app1.docx]

| Participants’ characteristics | | Groups | | | | | | Values | | | |
| --- | --- | --- | --- | --- | --- | --- | --- | --- | --- | --- | --- |
|  | | BioBase breathing (n=25), mean (SD) | | Mindfulness body scan (n=25), mean (SD) | | Control (n=25), mean (SD) | | *F* test (*df*) | | *P* value | η^2^ |
|  | | | | | | | | | | | |
| Age (years) | | 33.76 (10.20) | | 32.36 (11.3) | | 30.79 (9.9) | | 0.48 (2,72) | | .61 | .01 |
| **Mindfulness** | | | | | | | | | | | |
|  | Describe | 15.64 (2.17) | | 15.04 (2.44) | | 15.32 (1.99) | | 0.46 (2,72) | | .63 | .01 |
|  | Observe | 13.56 (4.48) | | 13.32 (4.16) | | 14.8 (3.93) | | 0.89 (2,72) | | .41 | .02 |
|  | Act-Aware | 12.80 (3.29) | | 12.38 (4.65) | | 14.04 (3.66) | | 1.10 (2,72) | | .33 | .03 |
|  | Non-judge | 13.56 (3.00) | | 14.16 (4.39) | | 15.88 (3.20) | | 2.81 (2,72) | | .06 | .07 |
|  | Non-react | 16.28 (3.43) | | 16.24 (3.21) | | 14.48 (3.66) | | 2.22 (2,72) | | .11 | .05 |
|  | Fatigue | 2.72 (1.30) | | 2.20 (1.19) | | 2.64 (1.03) | | 1.40 (2,72) | | .25 | .03 |
|  | Sleepiness/Alertness | 2.40 (0.86) | | 2.04 (1.02) | | 2.40 (0.86) | | 1.27 (2,72) | | .28 | .03 |
| **Baseline heart rate variability** | | | | | | | | | | | |
|  | RMSSD^a^ | 1.70 (0.22) | | 1.70 (0.29) | | 1.64 (0.30) | | 0.42 (2,72) | | .65 | .01 |
|  | pNN50^a^ | 1.21 (0.55) | | 1.30 (0.44) | | 1.06 (0.48) | | 1.42 (2,69) | | .24 | .04 |
|  | HF^a^ | 2.94 (0.58) | | 2.89 (0.67) | | 2.71 (0.43) | | 1.10 (2,72) | | .33 | .05 |
|  |  |  |  |  |  |  |  |  |  |  |  |
|  |  | Frequency |  | Frequency |  | Frequency |  | Df | x^2^ | *P* value | φ |
|  |  |  |  |  |  |  |  |  |  |  |  |
| Gender | |  |  |  |  |  |  |  |  |  |  |
|  | Male | 10 |  | 8 |  | 9 |  | 2 | 0.34 | .84 | .06 |
|  | Female | 15 |  | 17 |  | 16 |  |  |  |  |  |
| 31-40 hours per week | | 21 |  | 16 |  | 20 |  | 8 | 6.73 | .56 | .30 |
| Smoker | | 3 |  | 4 |  | 2 |  | 4 | 2.28 | .68 | .17 |
| High level of fitness | | 20 |  | 19 |  | 20 |  | 8 | 4.13 | .84 | .23 |
